# Sustainable Supramolecular Extraction of Phytocomplexes from Microgreens and Their Eco-Loading in Nutriosomes: Physicochemical Characterization, Stability, and In Vitro Release Behavior

**DOI:** 10.3390/molecules30183774

**Published:** 2025-09-17

**Authors:** Anja Vučetić, Rita Abi Rached, Maria Letizia Manca, Olja Šovljanski, Dragoljub Cvetković, Maria Manconi, Jasna Čanadanović-Brunet

**Affiliations:** 1 Department for Organic Chemistry, Faculty of Technology Novi Sad, University of Novi Sad, Bulevar cara Lazara 1, Novi Sad 21000, Serbia; 2 Department of Life and Environmental Sciences, University of Cagliari, University Campus, S.P. Monserrato-Sestu Km 0.700, 09042 Monserrato, CA, Italy; rita.abirached@unica.it (R.A.R.); mlmanca@unica.it (M.L.M.); manconi@unica.it (M.M.); 3 Department for Biotechnology, Faculty of Technology Novi Sad, University of Novi Sad, Bulevar cara Lazara 1, Novi Sad 21000, Serbia; oljasovljanski@uns.ac.rs (O.Š.); cveled@uns.ac.rs (D.C.)

**Keywords:** nutriosomes, supramolecular extraction, Brassicaceae microgreens, green nanotechnology, controlled release, gastrointestinal simulation

## Abstract

This study reports a dual green strategy for obtaining and stabilizing phytocomplexes from Sango radish and kale microgreens. Phytochemicals were isolated through supramolecular extraction, which generated an upper amphiphilic phase and a lower aqueous phase, enabling the recovery of both hydrophilic and lipophilic molecules without toxic solvents. The resulting phytocomplexes were encapsulated in nutriosomes, phospholipid vesicles enriched with the soluble dextrin Nutriose^®^ FM06, and compared with conventional liposomes. The vesicles displayed mean diameters ≤ 110 nm, polydispersity indices < 0.11, and zeta potentials around −40 mV. Retention of antioxidant activity reached up to 99%. Freeze-dried formulations maintained acceptable physicochemical properties and microbiological safety, while storage studies confirmed stability over six months. In vitro release tests showed a gradual release of phenolics and carotenoids, and simulated digestion experiments indicated that nutriosomes preserved up to 20% more antioxidant capacity than liposomes in the intestinal phase. These results demonstrate an environmentally responsible strategy to prepare phytocomplex-rich vesicles with improved stability and bioaccessibility. Further biological and in vivo studies are needed to substantiate potential nutritional or health-related benefits.

## 1. Introduction

Microgreens are grown from seeds lodged in high density on a suitable substrate and quickly harvested after germination and appearance of the first true leaves. They have a short growth cycle (7–21 days), and can be easily and sustainably grown in small areas, with low water consumption, by soilless cultivation and omitting fertilization and phytosanitary treatments [1]. These young edible plants, which are harvested at the cotyledon stage, have an intense flavor as well as high nutritional and phytochemical content, and particularly contain polyphenols, carotenoids, and glucosinolates [2]. Among various microgreens, the Brassicaceae families stand out because of their beneficial effects on human health [3]. Sango radish (*Raphanus sativus* var. *Sango*) and kale (*Brassica oleracea* var. *acephala*) are two Brassicaceae that have a significant content of phenolics, carotenoids, minerals, and vitamins, and are associated with a wide range of health benefits [4]. This profile makes them an ideal natural source for improving the nutritional quality of manufactured foods [5]. Two key steps are mandatory to pass from plants to enriching food ingredients: the extraction of phytocomplex and its formulation to ensure stability and bioavailability of phytochemicals [6].

Thus, the first challenge is the choice of extraction method, which significantly influences the yield and quality of the obtained phytocomplex. Conventional extraction techniques, such as solvent maceration or Soxhlet separation, have low selectivity and consummate high solvent volumes, and cause thermal degradation of chemicals [7]. In contrast, supramolecular extraction is an advanced and eco-friendly method that overcomes these inefficiencies as its extraction medium is composed of nanostructured liquids formed by colloid solutions made by self-assembling amphiphilic molecules [8]. These supramolecular aggregates, such as vesicles and micelles, create distinct microenvironments that solubilize hydrophilic and lipophilic molecules, making it possible to obtain in a single step a richer phytocomplex containing chemically diverse compounds [9].

One of the key advantages of supramolecular extraction is its high selectivity and efficiency in extracting phytochemicals. Unlike conventional solvents that dissolve all solutes indiscriminately, supramolecular extraction offers a tenable polarity gradient, enabling the precise selection of molecules based on hydrophobicity, charge, and molecular size [10]. This enhanced selectivity leads to higher extraction yields, minimizes the need for multiple purification steps, and enhances the cost-effectiveness for industrial applications. Another important advantage of this extraction method is its environmental sustainability, as biodegradable, non-toxic, and food-grade solvents are used, making the resulting material safe for food, nutraceutical, and pharmaceutical uses. In contrast, extraction with solvents requires the use of toxic organic solvents such as methanol, acetone, and chloroform, which have significant health and environmental hazards [11]. In particular, research regarding the extraction of microgreens has so far mostly used standard extraction analysis. This is especially true if nonpolar bioactive compounds are targeted. For example, the majority of the literature on carotenoids in microgreens describes extraction methods that use either acetone and water or hexane and hexane/toluene together with saponification [12]. The second important choice that affects the final fate and commercial value of food ingredients is their formulation. The loading in nanocarriers can notably improve the in vivo behavior of phytocomplexes, enhancing stability, release, bioavailability, and bioaccessibility [13]. Among various nanocarriers, liposomes and modified phospholipid vesicles are the most suitable for food formulation as they are mainly composed of naturally occurring phospholipids and water. They can encapsulate hydrophilic molecules and entrap lipophilic molecules into the bilayer, protecting the sensitive payloads from external environmental factors and delivering them through biological barriers [14]. Nutriosomes are advanced phospholipid nanovesicles tailored for oral use and enriched with a natural water-soluble dextrin (Nutriose^®^ FM06) that enhances stability and targeted delivery [15]. They were specifically designed to load antioxidant molecules, protect them from gastrointestinal fluids, improve their intestinal absorption, and modulate the enzymatic activity of gut microbiota [16].

This study aims to develop eco-sustainable food ingredients rich in antioxidants and carotenoids by integrating sustainable cultivation of Brassicaceae microgreens, eco-supramolecular extraction of their phytocomplexes, and nanoformulation into nutriosomes through a solvent-free sonication process.

## 2. Results

### 2.1. Extraction from Microgreens

**Figure 1:**
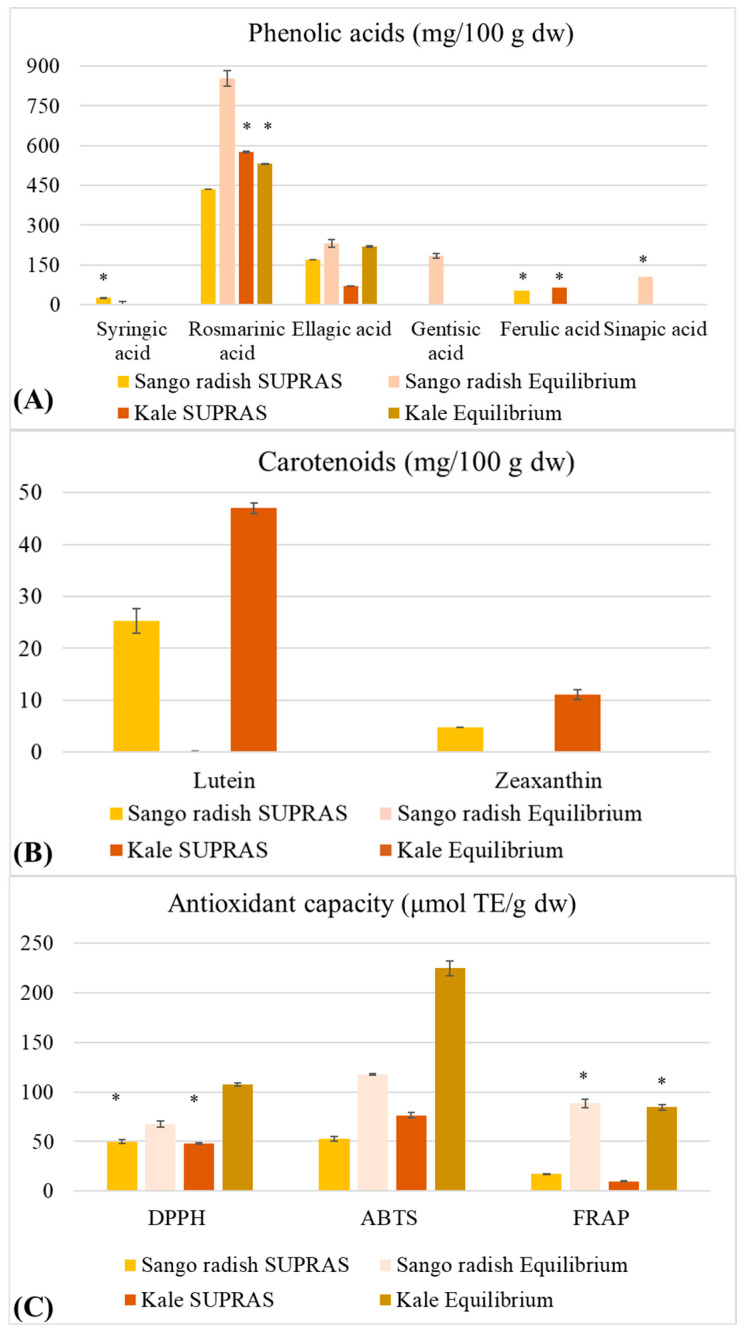
Content of phenolic acids (**A**), carotenoids (**B**), and antioxidant capacity (**C**) in extractive upper- and lower-phases from Sango radish and kale microgreens. Values are means ± SD (number of repetitions was 3). * Indicates significant difference at *p* < 0.05 (Tukey’s HSD test). Sango radish upper-phase = yellow, Sango radish lower-phase = orange, Kale upper-phase = red, Kale lower-phase = brown.

Unlike others, supramolecular extraction is a highly selective and efficient method that allows the simultaneous extraction of hydrophilic and lipophilic molecules only using food-grade solvents and low-dissipative processes to minimize environmental impact [10]. The composition of two obtained extractive solutions was evaluated by HPLC (Figure 1A,B) and antioxidant assays (Figure 1C).

Syringic acid was the least represented acid and was detected only in the two extract phases from Sango radish (~25 mg/100 g dried weight), while it was completely absent in the kale phases. Differently, ferulic acid (≈60 mg/100 g dried weight) was found in low amounts and only in the upper-phase of both microgreens. Gentisic acid and sinapic acid were detected only in the lower-phases of Sango radish (~183 mg/100 g dried weight) and kale (~104 mg/100 g dried weight), confirming the higher extractive performance of this phase versus polar molecules. Rosmarinic acid was the most prevalent phenolic acid in both phases of microgreens, especially in the lower-phase of Sango radish (~854 mg/100 g dried weight), compared to its upper-phase (~432 mg/100 g dried weight) and both extract phases of kale microgreens (≈574 mg/100 g dried weight). Ellagic acid was more abundant in the lower-phases of both microgreens (≈220 mg/100 g dried weight) and its lowest amount was present in the upper-phase of kale extract (~69 mg/100 g dried weight). In contrast to phenolic acids, carotenoids, which are nonpolar molecules, were exclusively extracted in the upper-phase, whereas they were not detected in the lower-phase. Around 25 mg/100 g dried weight of lutein was recovered in the upper-phase from Sango radish and ~47 mg/100 g dried weight in that from kale; ~5 mg/100 g dried weight of zeaxanthin was in the upper-phase from Sango radish and ~11 mg/100 g dried weight in that from kale. The antioxidant capacity of phytocomplexes obtained from Sango radish and kale microgreens was also measured using three different assays, DPPH, ABTS, and FRAP (Figure 1C). Each is based on a hydrogen atom transfer or a single electron transfer [17]. The obtained measurements were always higher in the lower-phase, irrespective of the test and microgreens used, according to their higher content of antioxidant phenolic acids.

### 2.2. Characterization of Vesicles

Thanks to their high versatility and double domain, the two extractive phases, containing the whole phytocomplex from Sango radish or kale microgreens, were simultaneously loaded in liposomes (used as reference) and nutriosomes. The mean diameter, polydispersity, zeta potential, and retention of antioxidant activity were measured (Table 1).

The mean diameter of liposomes loading both phytocomplexes was ≈ 101 nm, irrespective of the used microgreens. That of nutriosomes loading phytocomplex from kale was statistically equal, ~104 nm, and that of nutriosomes loading phytocomplex from Sango radish was slightly higher, ~110 nm, confirming that the soluble fiber did not affect the vesicle size. The polydispersity index of nutriosomes loaded with both phytocomplexes was very low, ~0.10; those of liposomes were slightly higher. The prepared liposomes and nutriosomes had negative zeta potential (≤−36 mV). Nutriosomes retained a higher amount of phytocomplexes than liposomes, as the retention of antioxidant activity (RAA%) of kale in nutriosomes was ~99%, and ~94% in liposomes.

**Figure 2:**
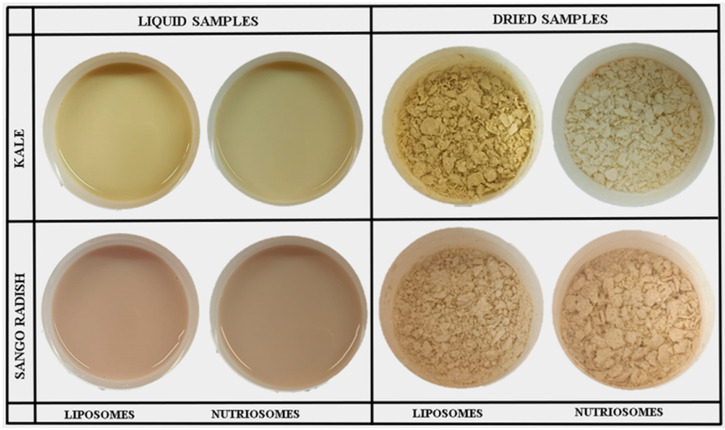
Macroscopic aspect of liquid dispersions and dried powders of liposomes and nutriosomes before and after freezer-drying.

Liposomes and nutriosomes were freeze-dried to facilitate and improve their storage stability and usefulness as food additives or dietary supplements (Figure 2).

The physicochemical characteristics of the powders of dried vesicles were measured (Table 2). Moisture content, which influences the stability and shelf-life of encapsulated powders, showed slight variations among the different formulations. Sango radish nanovesicles exhibited higher moisture levels, with nutriosomes containing 8.3 g/100 g and liposomes 7.9 g/100 g, compared to kale nanovesicles, where nutriosomes had 7.5 g/100 g and liposomes 7.1 g/100 g. Interestingly, liposomes and nutriosomes loading phytocomplex from kale showed higher dispersion stability, while nutriosomes loading Sango radish showed the lowest value at 50%, likely due to stronger interactions between the payloads and the phospholipids. Hygroscopicity, which measures the ability of powders to absorb water, was independent of the formulation. Flow properties, which impact the handling and drying process, were measured using the compressibility index and Hausner ratio. Lower compressibility and lower Hausner ratios generally indicate better flowability, which is advantageous in industrial applications. Nutriosomes loading phytocomplex from kale had the best flowability, a compressibility index of ~19%, and a Hausner ratio of ~1.2. In contrast, liposomes loading phytocomplex from kale had significantly higher compressibility index (~47%) and a Hausner ratio of ~1.9, indicating poorer flow characteristics. Similar trends were observed in vesicles loading phytocomplex from Sango radish formulations as nutriosomes (compressibility index: ~21%, Hausner ratio: ~1.3) had better flow properties than liposomes (compressibility index: ~46.7%, Hausner ratio: ~1.9). The color properties of vesicles are important for their potential applications in functional foods and nutraceuticals. Differences in lightness (L*), red–green balance (a*), yellow–blue balance (b*), and saturation (C*) suggested that nutriosomes allowed retention of pigments and structural integrity differently than liposomes.

### 2.3. Stability of Vesicles over 6 Months

**Figure 3:**
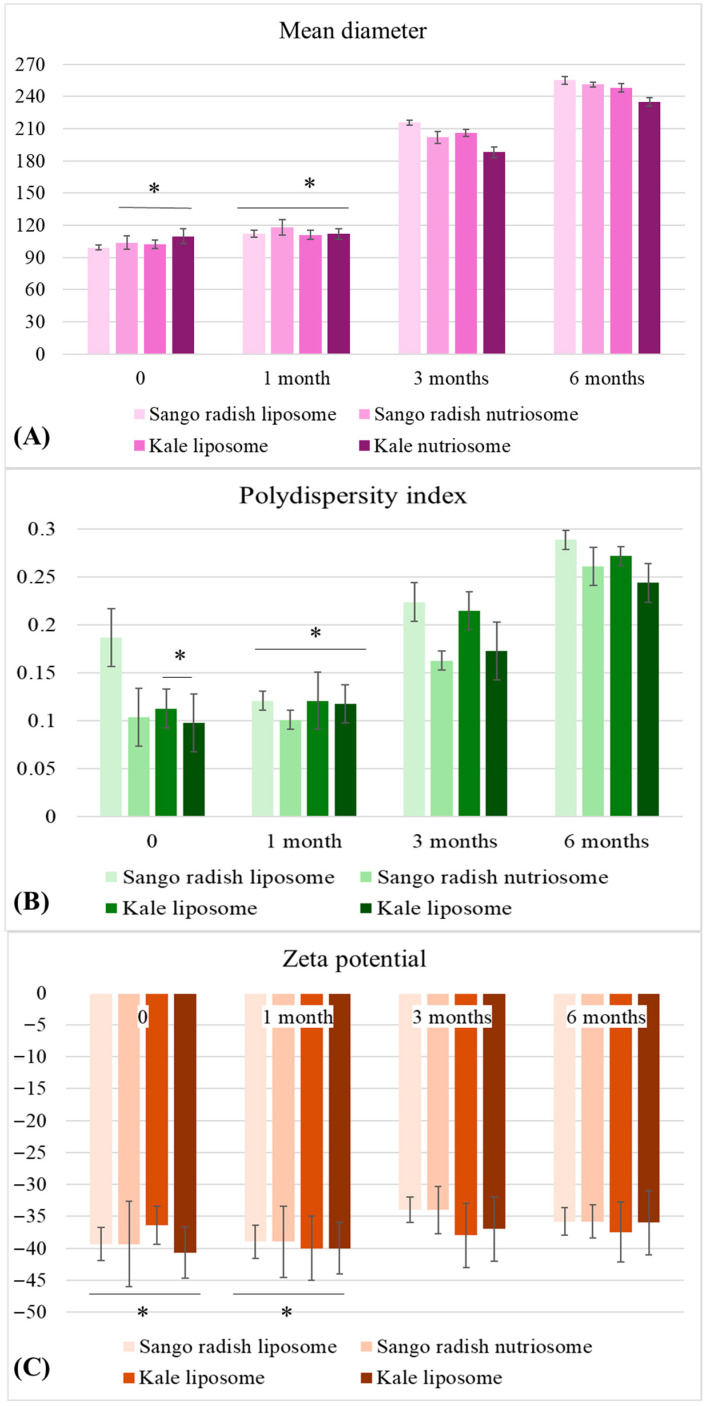
Change in mean diameter (**A**), polydispersity index (**B**), and zeta potential (**C**) of liposomes and nutriosomes during 6 months. Values are means ± SD (n = 3). * Indicates significant difference at *p* < 0.05 (Tukey’s HSD test).

The liposomes and nutriosomes were stored, and their mean diameter, polydispersity index, and zeta potential were measured to evaluate their stability over 6 months (Figure 3).

After one month, there was a noticeable increase in mean diameter, with all formulations expanding to sizes between 111 nm and 118 nm. By month three, a more pronounced increase was observed, particularly in Sango radish liposomes (215 nm) and kale liposomes (206 nm). Finally, after six months, all formulations showed more significant growth, with Sango radish liposome reaching 289 nm. Meanwhile, the polydispersity index (PI) values increased over time, reflecting structural instability and particle aggregation. By month six, Sango radish liposomes had the highest PI (0.29), indicating higher heterogeneity. Up to three months, Sango radish nutriosomes (0.16) and kale nutriosomes (0.17) maintained relatively better stability, while at the six-month mark, PI diversity rose to 0.26 and 0.24, respectively. For zeta potential, after one month, values remained stable, fluctuating between −39 mV and −42 mV, indicating well-dispersion and resistance to aggregation. Sango radish liposomes showed the lowest zeta potential (−34 mV), representing reduced colloidal stability compared to nutriosomes (−40 mV).

### 2.4. In Vitro Release of Phenolics and Carotenoids from Vesicles

**Figure 4:**
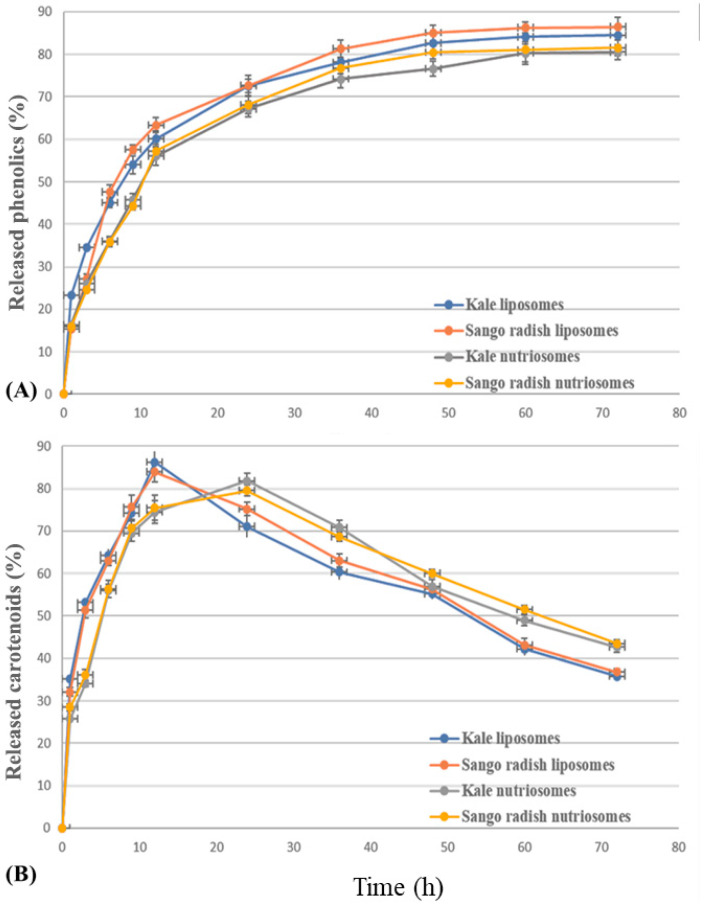
Amount of phenolics (**A**) and carotenoids (**B**) released from liposomes and nutriosomes loading phytocomplex form Sango radish and kale microgreens. Mean values ± standard deviations are reported.

The amount of phenols and carotenoids released from liposomes and nutriosomes was measured (Figure 4). The amount of phenolics released from liposomes increased quickly up to ~61% at 9 h, and that of nutriosomes up to ~56% at 11 h, after which, it increased slowly but constantly up to ~80% (liposomes) and ~70% (nutriosomes) at 50 h. Finally, the phenolic amount reached a plateau and remained constant (~90%). The release profiles of carotenoids were different from those of phenols because the carotenoid amount increased quickly up to ~85% from liposomes at 12 h and ~80% from nutriosomes at 22 h. Following this peak, the curves decreased, and the profile of liposomes and nutriosomes was inverted, as, at 72 h, the carotenoid amount was ~37% from liposomes and ~45% from nutriosomes, probably due to their partial degradation in solution.

### 2.5. Antioxidant Capacity of Vesicles in Simulated In Vitro Digestion

**Figure 5:**
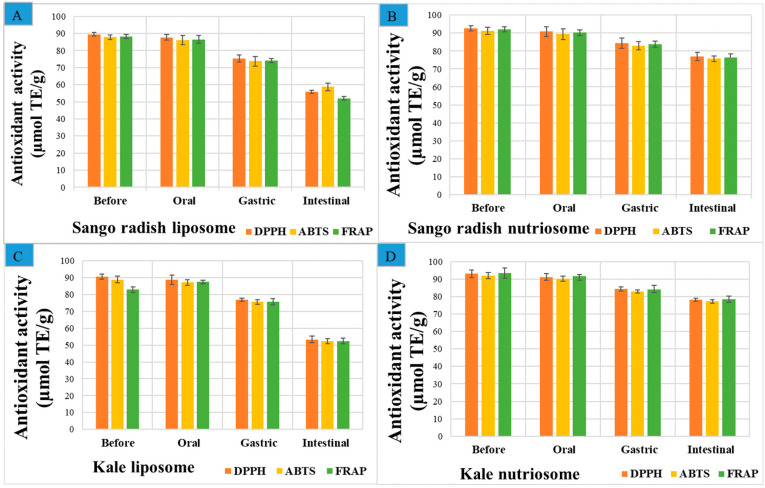
Antioxidant capacity of liposomes (**A**,**C**) and nutriosomes (**B**,**D**), loading phytocomplex from Sango radish and kale, measured before and during in vitro digestion. Mean values ± standard deviations are reported.

A simulated gastrointestinal digestion model, mimicking the physiological conditions of the human digestive apparatus [18], was used to evaluate the post-digestion bioaccessibility of phytocomplexes loaded in vesicles. The effectiveness of the liposomes and nutriosomes in preserving the antioxidant capacity of loaded phytocomplexes during digestion was measured (Figure 5). The antioxidant capacity was measured using DPPH, ABTS, and FRAP assays. The results obtained with the three tests were perfectly superposable.

Before digestion, the antioxidant capacity of nutriosomes was higher than that of liposomes, due to their higher antioxidant retention efficiency. Specifically, nutriosomes loading phytocomplex from kale had the highest initial antioxidant capacity, corresponding to ~93 µmol Trolox equivalents/g dried weight with DPPH, ~92 µmol Trolox equivalents/g dried weight with ABTS, and ~94 µmol Trolox equivalents/g dried weight with FRAP. Conversely, liposomes loading phytocomplex from Sango radish had the lowest values, ~90 µmol Trolox equivalents/g dried weight with DPPH, ~88 µmol Trolox equivalents/g dried weight with ABTS, and ~88 µmol Trolox equivalents/g dried weight with FRAP. As digestion progressed to the gastric phase, a more noticeable decline in antioxidant capacity was observed, and that of phytocomplexes loaded in liposomes was greater than that loaded in nutriosomes. This phase involved exposure to acidic conditions (pH 3.0) and pepsin enzymes, which contributed to polyphenol oxidation and structural breakdown. The antioxidant capacity of liposomes loading phytocomplex from kale decreased from ~90 to ~77 µmol Trolox equivalents/g dried weight measured with DPPH (−15%), whereas that of corresponding nutriosomes was higher ~84 µmol Trolox equivalents/g dried weight (−10%). The antioxidant capacity of liposomes loading phytocomplex from Sango radish dropped from ~89 to ~75 µmol Trolox equivalents/g dried weight with DPPH (−16%), while that of corresponding nutriosomes decreased less than ~84 µmol Trolox equivalents/g dried weight (−8%). The most significant decline occurred during the intestinal phase. The antioxidant capacity of liposomes loading phytocomplex from kale decreased from ~90 to ~53 µmol Trolox equivalents/g dried weight (−41%) with DPPH, whereas that of corresponding nutriosomes was ~78 µmol Trolox equivalents/g dried weight (−16%). The antioxidant capacity of liposomes loading phytocomplex from Sango radish decreased from ~89 to ~56 µmol Trolox equivalents/g dried weight (−37.6%), and that of nutriosomes was ~77 µmol Trolox equivalents/g dried weight (−17%).

### 2.6. Microbiological Content of Vesicles

The microbiological contamination of formulations is another important aspect that must be evaluated to design food ingredients to ensure consumer health and regulatory compliance [19]. The presence of aerobic bacteria, *Enterobacteriaceae*, yeasts, fungi, *Salmonella* spp., and sulfite-reducing clostridia was evaluated in phytocomplexes loaded in liposomes and nutriosomes (Table 3).

The levels of total aerobic bacteria, *Enterobacteriaceae,* yeasts, and fungi were below the detectable limit (<1 log CFU/g or mL), confirming that the extraction methods used and the loading of phytocomplex in liposomes and nutriosomes effectively prevent microbial contamination. Notably, *Salmonella* spp. was not detected in any sample, confirming that formulations meet food safety criteria. The absence of sulfite-reducing clostridia (<1 log CFU/g or mL) further supports their suitability for food and nutraceutical applications [19,20].

## 3. Discussion

### 3.1. Extraction from Microgreens

The results support the high selectivity and efficiency of supramolecular extraction in isolating both polar and nonpolar compounds from microgreens using green solvents, along with the reduced consumption of solvents [21]. The lower-phase seems more suitable for extracting polar organic acids; indeed, ellagic, gentisic, and sinapic acids, which are highly polar, were only found in this phase [22]. Nevertheless, different phenolic acids and carotenoids were found in the extractive solutions as a function of the phase and the used microgreens, thanks to the selectivity of the used method. Supramolecular phases are engineered to contain both hydrophobic and hydrophilic domains, creating internal microenvironments with distinct chemical affinities [23]. The results of this research indicated that the lower-phase predominantly accumulated hydrophilic components, whereas the upper-phase enabled the simultaneous extraction of both polar phenolics and nonpolar carotenoids. The extraction of carotenoids exclusively in the upper supramolecular phase is also supported in the research of Travičić et al. [24].

In general, previous studies confirmed the high antioxidant capacity of Brassicaceae microgreens [25,26,27]. While similar activity was presented in both upper-phase extracts, the scavenging activities measured by DPPH and ABTS were notably higher in the Sango radish lower-phase, which can be mainly related to the presence of ellagic and rosmarinic acids. In contrast, kale microgreens showed a markedly higher reducing power in the upper-phase (17.58 µmol TE/g dw) compared to Sango radish (9.09 µmol TE/g dw), as demonstrated by the FRAP assay. A correlation between kale’s higher carotenoid content and FRAP activity could be drawn, given that carotenoids have been noted to possess limited DPPH activity, but a significant FRAP potential by the research of Müller et al. [28].

This extraction method, coupled with the rich phytocomplex of microgreens, opens new opportunities to develop nature-based future foods, especially if adequately formulated in advanced systems [29].

### 3.2. Characterization of Vesicles

Characterization of liposomes and nutriosomes demonstrated that both systems formed nanoscale, stable vesicles with good colloidal properties. Mean diameter is a critical parameter influencing stability, bioavailability, and dispersion properties of vesicles, and the fact that loaded nutriosomes remained below 110 nm indicates favorable characteristics for their application. The polydispersity index reflects the uniform distribution of vesicles, and values ≤ 0.3 confirm the formation of uniform colloidal dispersions; meanwhile. Both types of nutriosomes possessed PI around 0.1, indicating monodispersed particles. The value of zeta potential indicates the surface charge of nanoparticles, which can positively affect their electrostatic repulsion and stability in dispersion. Usually, high absolute values (greater than 30 mV) indicate better colloidal stability with reduced likelihood of aggregation [30]. The prepared liposomes and nutriosomes had negative zeta potential (≤−36 mV) due to the predominant negative charge of phosphatidyl choline at neutral pH. Namely, the retention of antioxidant activity of payloads in carriers is a key parameter of their delivery power, and ensures effective loading and protection of bioactive compounds [31]. In this study, the RRA of Sango radish phytocomplex into both nutriosome and liposome (~96 and ~91%) was slightly lower in both nutriosomes (~96%) and liposomes (~91%), compared to that prepared with kale extracts (~99% and ~94%, respectively). This variance is likely attributed to the presence of anthocyanidins in Sango radish, which are known to be structurally less stable and more sensitive to processing conditions [32].

Retention of antioxidant activity in Sango radish nutriosomes was ~96% and ~91% in liposomes, indicating the special suitability of nutriosomes as an effective system to load, protect, and deliver the natural payloads. Teli et al. [33] have demonstrated that nutriosomes, which integrate phospholipids with bioenhancers, permit an increase in the antioxidant retention activity, accordingly.

Liposomes and nutriosomes loading phytocomplex from Sango radish had a higher moisture content due to an incomplete drying process, which can reduce their stability. Solubility is a critical parameter that allows for the evaluation of the bioavailability of payloads. Even though liposomes and nutriosomes are not molecularly soluble in water, their ability to remain dispersed in aqueous medium was investigated. Flow properties, which impact the handling and processing of encapsulated powders, were evaluated using the compressibility index and Hausner ratio. Lower compressibility and lower Hausner ratios generally indicate better flowability, which is advantageous in industrial applications. The superior flowability of nutriosomes may be attributed to the presence of the soluble dextrin (Nutriose^®^), which is an extremely fine and flowing powder that can reduce interparticle friction and improve powder handling. Additionally, it can act as a cryoprotectant, preventing the breakage of vesicles during the drying process and allowing the formation of more stable, free-flowing powders, which can be used in large-scale production [34]. These findings are consistent with studies on phospholipid delivery systems, which highlight the role of composition in improving powder flow [35].

The lightness indicates that nutriosomes loading phytocomplex from kale tended to be slightly brighter than corresponding liposomes. In contrast, nutriosomes loading phytocomplex from Sango radish were less bright than liposomes, possibly due to interactions between phospholipids and anthocyanins. The red–green balance revealed that nutriosomes loading phytocomplex from Sango radish and kale had a similar green that shifted toward neutrality, suggesting potential anthocyanin instability, while corresponding liposomes had a moderate green hue. The yellow–blue balance of nutriosomes loading phytocomplex from Sango radish indicated an enhanced retention of yellow carotenoids. The yellow–blue balance of nutriosomes loading phytocomplex from kale was significantly higher compared to that of liposomes, indicating improved carotenoid stability. Moreover, the saturation, which reflects color intensity, further differentiates the formulations. The yellow–blue balance of nutriosomes loaded with phytocomplexes from Sango radish and kale showed higher saturation compared to liposomes, suggesting that nutriosomes not only retain but also enhance the chromaticity of the encapsulated pigments. Overall, these results imply that while nutriosomes had superior brightness, yellowness, and color intensity, beneficial for visually appealing functional foods, they may require further optimization to fully preserve anthocyanin stability. Overall, the results further emphasized that nutriosomes outperform liposomes in retention of antioxidant activity, powder flowability, brightness, yellowness, and color intensity, making them a promising formulation for food. While encapsulation efficiencies up to 99% were observed, it should be noted that these values are based on antioxidant capacity as an indirect proxy. Future studies, including direct quantification of encapsulated compounds by HPLC after dialysis, would further strengthen the reproducibility and accuracy of EE% values.

### 3.3. Stability of Vesicles over 6 Months

The trend shown in Figure 3 suggests that nutriosomes exhibited slightly better stability over time, possibly due to their improved resistance to particle aggregation and degradation. The increase in mean diameter over time is consistent with findings in the literature, which report that liposomal vesicles tend to fuse and form larger aggregates due to vesicle instability, leading to compromised encapsulation efficiency [36,37]. In contrast, nutriosomes, phospholipid vesicles enriched with bioenhancers, appear to better maintain their structural integrity, preventing excessive growth and aggregation.

Sango radish liposomes showed the lowest zeta potential (−34 mV), representing reduced colloidal stability compared to nutriosomes (−40 mV). This suggests that nutriosomes provide better stability due to their enhanced lipid interactions, preventing vesicle fusion and precipitation. For zeta potential, after one month, values remained stable, fluctuating between −39 mV and −42 mV, indicating well-dispersion and resistance to aggregation. Sango radish liposomes showed the lowest zeta potential (−34 mV), representing reduced colloidal stability compared to nutriosomes (−40 mV). This suggests that nutriosomes provide better stability due to their enhanced lipid interactions, preventing vesicle fusion and precipitation.

These results align with previous studies, which highlighted that nutriosomes exhibit stronger electrostatic repulsion and lipid integrity, thereby delaying aggregation and degradation over extended storage periods [30]. Nutriosomes generally display superior stability due to their optimized lipid composition, as demonstrated by Paul et al. [38]. Even though all formulations maintained acceptable parameters during their six-month storage, with nutriosomes providing slightly more controlled and homogeneous encapsulation, the noticeable increase in mean diameter and polydispersity index (PI) from the third month onward raises concerns about their long-term stability. An increase in mean diameter over time is expected due to aggregation and structural rearrangements, as noted by Patel [37]. However, incorporating stabilizing polymers could be a promising strategy to enhance their stability further [39,40]. Zeta potential plays a crucial role in preventing aggregation, and maintaining values below −30 mV is critical for long-term stability [36]. From an application standpoint, these outcomes reinforce that nutriosomes are a more promising encapsulation system for bioactives requiring long-term stability, particularly in functional food, nutraceutical, and pharmaceutical formulations, where the integrity of the nanocarrier system is essential. The noticeable particle size increase in liposomes suggests aggregation, which is consistent with their lower zeta potential and less uniform dispersion compared to nutriosomes. A limitation of the present study is the absence of direct ultrastructural confirmation by TEM or SEM, which will be considered in future investigations to complement the stability assessment. Although stability evaluation was based on dynamic light scattering parameters, future studies should include direct morphological imaging to confirm structural changes during storage. Furthermore, the use of cryoprotectants or stabilizing polymers during freeze-drying may further enhance the stability of vesicles, and this optimization is planned for our next investigations.

### 3.4. In Vitro Release of Phenolics and Carotenoids from Vesicles

The in vitro release studies established that nutriosomes release phenolics and carotenoids more slowly than liposomes, and better retain carotenoids over prolonged periods.

The release curves of phenolics from liposomes and nutriosomes loaded with both phytocomplexes were extremely similar; however, those of nutriosomes were consistently lower, indicating a slower release. The degradation curve of loaded carotenoids showed a different pattern. Liposomes and nutriosomes reached their peak release at around 15 h and 24 h, respectively, after which significant degradation of the released carotenoids was observed. This can be attributed to their sensitivity to light, temperature, and pH, which was extensively observed in studies in the literature [41].

Such controlled release behavior can enhance the functional performance of antioxidant compounds in foods, as demonstrated in this case. Interestingly, nutriosomes not only delayed the release but also protected against environmental factors, allowing a higher concentration to be preserved at 72 h. These benefits were conferred by the soluble dextrin, as previously reported for curcumin [15].

### 3.5. Antioxidant Capacity of Vesicles in Simulated In Vitro Digestion

Simulated digestion experiments validated the stability and bioaccessibility of loaded phytocomplexes. The oral digestion phase led to minimal reductions (~2%) in antioxidant capacity across all samples, probably since α-amylase, the primary enzyme in saliva, does not significantly degrade polyphenols or carotenoids. At this stage, all formulations retained over ≈87% of their initial antioxidant capacity, with slight reductions observed, likely due to mechanical interactions and minor solubilization of polyphenols rather than enzymatic degradation. Results suggested that early-stage digestion does not compromise the integrity of phytochemicals, reinforcing their potential for nutraceutical applications.

The superior protection provided by nutriosomes in gastric digestion may be attributed to the presence of Nutriose^®^, which is usually partitioned preferably to one of the phospholipid leaflets, reinforcing the vesicle structure [15]. In addition, Borges et al. [29] suggested that nutriosomes may mitigate the impact of gastric conditions on the degradation of polyphenols. The intestinal digestion phase caused the most significant reductions in antioxidant capacity of the samples, primarily due to the activity of pancreatic enzymes and interactions with bile salts, which degraded phenols. Liposomes continued to show greater losses compared to nutriosomes, reinforcing the superior stability of nutriosome vesicles. The antioxidant capacity of both phytocomplexes loaded in nutriosomes decreases less, especially during gastric and intestinal digestion, confirming the ability of these vesicles to protect the antioxidant payloads and control their release. The higher proportion of bioactive compounds in the intestine suggests greater bioaccessibility and absorption potential. Phytocomplexes loaded in nutriosomes had a higher antioxidant capacity than liposomes after preparation, were minimally degraded (~2%) in the oral phase, and were better protected in the gastric digestion and intestinal phase. Thus, their stability and bioaccessibility were potentially improved. Borges et al. [29] reported that nutriosomes may enhance bioavailability due to their improved interaction with biological membranes, accordingly. Given their superior performances, nutriosomes represent a more effective delivery system to prepare antioxidant ingredients for functional foods, nutraceuticals, and dietary supplements. As a specific note for these experiment steps, some limitations can be observed. Since this study omitted gastric lipase and shortened the gastric phase (vs. INFOGEST 2.0), absolute gastric-phase carotenoid release may be underestimated, whereas polyphenol stability may be relatively over-conserved; therefore, the used digestion outcomes are best interpreted as within-protocol comparisons between nutriosomes and liposomes under the same conditions.

Our digestion outcomes confirm enhanced in vitro stability and bioaccessibility of nutriosome-loaded phytocomplexes, but the actual nutritional efficacy must be validated by cellular uptake and absorption studies.

### 3.6. Microbiological Content of Vesicles

Regulatory agencies such as the European Food Safety Authority (EFSA) and the U.S. Food and Drug Administration (FDA) emphasize that microbiological assessment is a key factor in approving novel food ingredients [42]. Microbiological analysis confirmed that the applied methods, extraction, formulation into vesicles, and freeze-drying, maintained microbiological safety. No total mesophilic bacteria, coliforms, yeasts, or molds growth was observed in freeze-dried samples during storage, with values consistently below the detection limit of 10 CFU/g (ISO 4833-1:2013). These results comply with microbiological safety criteria for powdered food ingredients (EU Commission Regulation (EC) No 2073/2005), where thresholds for these microorganisms range between 10^2^ and 10^3^ CFU/g depending on the product category. Therefore, the formulations can be considered microbiologically safe under the tested conditions.

## 4. Materials and Methods

### 4.1. Microgreens, Chemicals, and Equipment

Microgreens from the Brassicaceae family, kale (*Brassica oleracea* var. *sabellica*) and Sango radish (*Raphanus sativus* var. *Sango*) were cultivated under controlled greenhouse conditions and harvested at their peak nutrient density (7 days after germination) as previously reported by Vučetić et al. [3]. Lipoid S75, a mixture of soybean phospholipids (70% phosphatidylcholine, 9% phosphatidylethanolamine, and 3% lysophosphatidylcholine), triglycerides, and fatty acids, was purchased from Lipoid GmbH (Ludwigshafen, Germany). Nutriose FM06^®^, a soluble dextrin from maize, was provided by Roquette (Lestrem Cedex, Beinheim, France). Standards of identified phenolic acids and carotenoids were purchased from Sigma Aldrich Chemie GmbH (Taufkirchen, Germany). At the same time, ethanol, methanol, and other reagents and solvents of analytical grade were obtained from Lach-Ner (Neratovice, Czechia).

### 4.2. Supramolecular Extraction of Phytocomplex from Microgreens

Phytocomplex was isolated by supramolecular extraction using parameters obtained in a previous optimization study [9]. Before the extraction method, the supramolecular phase was prepared using 36% ethanol, 5% octanoic acid, and distilled acidified water (pH 3), vortexing the mixture for 1 min and centrifuging it at 4000 rpm (Gramma Libero LACE 24, Belgrade, Serbia) before phase separation. Besides the amphiphilic supramolecular phase, known in the literature as SUPRAS, the equilibrium was also obtained, used for further extraction, and loaded into the phospholipod vesicles. For clarity, the SUPRAS phase will hereafter be referred to as the upper-phase, while its equilibrium counterpart will be referred to as the lower-phase. The mass of 30 mg of dried plant material was transferred to 1 mL of a biphasic solvent system containing upper-phase and lower-phase in a 1:1 (*v*/*v*) ratio. Sequential treatments were performed in an ultrasonic bath and a laboratory shaker for 15 min each, followed by centrifugation at 4000 rpm for 20 min. After extraction, ethanol was removed using a rotary vacuum evaporator at 50 °C (Rotavapor RII, BÜCHI Labortechnik AG, Flawil, Switzerland) under reduced pressure. The obtained two phases were then separated and stored at −4 °C until further use.

### 4.3. HPLC Analysis of Phytocomplexes

The phenolic and carotenoid profiles of phytocomplexes were analyzed using a Shimadzu Prominence HPLC equipped with an LC-20AT binary pump, CTO-20A thermostat, and SIL-20A autosampler with a DAD detector obtained by Shimadzu (Kyoto, Japan). Both supramolecular phases (30 mg/mL) were diluted in mobile phases (1:1) and filtered before analysis. The separation of phenolic acids was performed using a Luna C-18 RP column, 5 mm, 250 × 4.6 mm (Phenomenex, Torrance, CA, USA) with a C18 guard column, 4 × 30 mm (Phenomenex). Elution was carried out using two mobile phases: acetonitrile (A) and 0.1% formic acid in water (B). The gradient profile started from 10% to 25% B in the first 10 min, followed by a linear increase from 10 to 20 min up to 60% B, and from 20 to 30 min up to 70% B. The gradient then returned to the initial condition of 10% B, with an additional 5-min equilibration time. Detection was performed at 280 and 320 nm, with a flow rate of 1 mL/min and an injection volume of 20 µL. Carotenoids were identified with reverse-phase HPLC analysis using a PerkinElmer Quasar C18 column, 250 × 4.6 mm, 5 μm (PerkinElmer, High Wycombe, Buckinghamshire, UK). Isocratic elution was performed with methanol: tetrahydrofuran stabilized with 0.1% butylated hydroxytoluene (95:5, *v*/*v*) as a mobile phase, at a flow rate of 1 mL/min, injection volume of 20 µL, 36 °C, and detection wavelength set at 445 nm. Calibration curves were obtained using standard solutions (r^2^ > 0.99) and used to quantify the phytochemicals. All data acquisition was relayed with LC Solution Software version 1.25 (Shimadzu, Kyoto, Japan), and final results were expressed as mg/100 g dry weight of plant material. Calibration curves were obtained using standard solutions (0.01–100 μg/mL) for each identified compound, and equations with regression coefficients are reported in Supplementary Table S1.

### 4.4. Quantification of Carotenoids, Phenols, and Antioxidants of Phytocomplexes

The microwell-adapted spectrophotometer Multiscan GO (Thermo Fisher Scientific Inc., Waltham, MA, USA) was used to quantify carotenoids and phenolics that were further utilized to assess the in vitro release of bioactive compounds from the vesicles. The content of total carotenoids was determined by direct measurement of diluted samples at 663, 645, 515, and 453 nm absorbances and calculated as mg of β-carotene/100 g dry weight, as per the method of Nagata and Yamashita [43], and adapted for a 96-well microplate. The results were derived using Equation (1).

C _(mg β-carotene/100mg)_ = 0.216 × A_663nm_ − 1.22 × A_645nm_ − 0.304 × A_505nm_ + 0.452 × A_453_ (1)

To measure total phenolics, diluted sample, distilled water, Folin–Ciocalteu reagent, and 20% sodium carbonate solution were mixed and, after 1 h, the absorbance was read at 750 nm with a 96-well microplate [44]. Phenol content was calculated using gallic acid as a reference and expressed as mg of gallic acid/100 g dry weight of plant material. Antioxidant capacity of the phytocomplexes was measured with the DPPH (2,2-diphenyl-1-picrylhydrazyl), ABTS (2,2′-azino-bis (3-ethylbenzothiazoline-6-sulfonic acid)), and FRAP (Ferric Reducing Antioxidant Power) assays. Briefly, samples were mixed with a 0.89 mM methanolic DPPH solution, and the absorbance was recorded at 515 nm after 50 min. Similarly, samples were added to the prepared ABTS solution, and the absorbance was measured at 414 nm after incubation at 27 °C for 35 min [3]. The FRAP working solution and the samples were mixed and incubated for 10 min at 37 °C, according to the method of Benzie et al. [44]. The absorbances were then recorded at 593 nm. Results were expressed as µmol of Trolox equivalents per g of dried weight.

### 4.5. Preparation of Nutriosomes Loading Phytocomplexes

The two extraction phases (15 mg/mL each) were mixed with phospholipid S75 (90 mg/mL) and Nutriose^®^ FM (60 mg/mL), and hydrated with 2 mL of distilled water. Dispersions were then sonicated using a Soniprep 150 sonicator (MSE Crowley, London, UK) at 30 cycles (5 s on and 2 s off) with a 12 μm probe amplitude. Liposomes without Nutriose^®^ FM were prepared and used as a reference. After preparation, liquid samples were freeze-dried in a Martin Crist Alpha freeze-dryer (Osterode am Harz, Germany) for 48 h, at −40 °C and 0.01 bars, and stored at 4 °C until use.

### 4.6. Characterization of Vesicles

The mean diameter, polydispersity index, and zeta potential, as indicators of homogeneity and colloidal stability, were measured using a Zetasizer Ultra (Malvern Instruments, Worcestershire, UK). Before the analysis, each sample was diluted 1:100 with distilled water to ensure optical clarity [45]. The long-term stability was evaluated by storing the dispersions at room temperature for six months and periodically measuring (one, three, and six months) the mean diameter, polydispersity index, and zeta potential.

Retention of antioxidant activity was determined after purification of vesicle dispersions by dialysis. One milliliter of vesicle dispersion was placed into a Spectra/Por^®^ dialysis membrane (12–14 kDa cut-off, 3 nm pore size; Spectrum Labs, Breda, The Netherlands) and immersed in 1 L of distilled water under constant stirring for 2 h at room temperature to remove unentrapped compounds. The antioxidant capacity of the sample was measured before and after dialysis using the DPPH assay (Section 4.4). RAA% was calculated according to the following Formula (2).

RAA% = (AA% _after dialysis_/AA% _before dialysis_) × 100 (2)

The results indicate the proportion of antioxidant capacity retained in the vesicles post-purification.

### 4.7. Evaluation of Physicochemical Properties of Freezer-Dried Vesicles

The moisture content, water activity, bulk density, tapped density, Carr index, and Hausner ratio of the powders were measured [46]. To calculate the moisture content, samples were further dried in an air oven at 105 °C until a constant weight. Water activity was measured by storing the samples in an airtight plastic container over a NaCl-saturated solution at 25 °C for 7 days. The value was calculated from the ratio of the initial weight of the samples versus that after the storage. Bulk and tapped densities were estimated by measuring the volume of samples in a graduated cylinder before and after tapping (250 times). These values were used to evaluate the flowability and cohesiveness of powders, calculating the Carr index and the Hausner ratios. Color analysis (CIE Lab method) was performed with a Minolta ChromaMeter CR-300 reflectance colorimeter (Minolta, Osaka, Japan) based on the CIELab color system. Chroma (or saturation) was calculated using the following equation: C* = √(a^2^ + b^2^).

### 4.8. Evaluation of In Vitro Digestibility and Bioaccessibility of Vesicles

The post-digestion bioaccessibility of samples was evaluated using a static INFOGEST protocol adapted from Brodkorb et al. [47] with the following pre-specified deviations tailored to vesicular, low-fat dispersions and antioxidant-focused endpoints: (i) gastric lipase was not included; (ii) the oral phase was 5 min (vs. 2 min in INFOGEST 2.0) to ensure homogeneous mixing of viscous dispersions; and (iii) the gastric phase was 60 min (vs. 120 min) to reduce artifactual oxidative degradation of labile phytochemicals during prolonged acid exposure. Operationally, samples were as follows: (oral) mixed with artificial saliva containing 75 U/mL α-amylase at 37 °C for 5 min; (gastric) incubated with 2000 U/mL pepsin at pH 3.0 and 37 °C for 60 min; and (intestinal) incubated with pancreatin (100 U/mL trypsin activity) and bile salts (10 mM) at pH 7.0 and 37 °C for 120 min. The antioxidant capacity of pre- and post-digestion samples was analyzed with DPPH, ABTS, and FRAP assays (Section 4.4).

The post-digestion bioaccessibility of samples was evaluated using a simulated gastrointestinal digestion model (INFOGEST in vitro model) [47]. To simulate the digestion, samples were as follows: (1) (oral phase) mixed with artificial saliva containing 75 U/mL of α-amylase and incubated at 37 °C for 5 min; (2) (gastric phase) incubated with 2000 U/mL of pepsin for 60 min at 37 °C and pH 3.0; and (3) (intestinal phase) digested with pancreatin (100 U/mL trypsin activity) and bile salts (10 mM final concentration) for 120 min at pH 7.0. The antioxidant capacity of pre- and post-digestion samples was analyzed with DPPH, ABTS, and FRAP assays (Section 4.4).

### 4.9. In Vitro Release of Carotenoids and Phenols from Vesicles

The amount of carotenoids and phenols in vitro released from vesicles was measured according to Kumar et al. [48]. Freezer-dried samples (0.1 g) were re-dispersed in 1 mL of 0.01 mol/L phosphate-buffered saline solution at pH 7.4 and incubated at 37 °C for 74 h. Aliquots of solution were withdrawn at scheduled time points (0, 1, 3, 6, 9, 12, 24, 36, 48, 60, 72 h). The samples were diluted in hexane and methanol, and the content of carotenoids and phenols was measured (Section 4.4). The percentage of released phytochemicals was calculated using Equation (3).

Release (%) = (Released phytochemicals)/(Total phytochemicals) × 100(3)

### 4.10. Measurement of Microbiological Content of Vesicles

Total aerobic bacteria [49], *Enterobacteriaceae* [50], yeasts and fungi [51], were counted and reported as log of colony-forming units versus g or mL of dispersion. The presence or absence of *Salmonella* spp. [52] and sulphite-reducing clostridia [53] were also assayed.

### 4.11. Statistical Analysis

All the experiments were performed in triplicate, and the mean value ± standard deviation was reported. Statistical analysis was performed to determine significant differences among the four experimental groups. One-way analysis of variance (ANOVA) was applied to each parameter to assess whether statistically significant differences existed among the means. When a considerable F-ratio was observed (*p* < 0.05), the Tukey Honestly Significant Difference (HSD) post hoc test was employed to identify which pairwise comparisons differed significantly. Statistical equivalence between groups was denoted using asterisk symbols (*), indicating values that were not significantly different (*p* > 0.05). All statistical calculations were performed using Statistica version 13.3.

## 5. Conclusions

This work demonstrates that combining sustainable supramolecular extraction with nutriosome encapsulation enables the preparation of microgreen-derived phytocomplexes that are rich in phenolics and carotenoids, colloidally stable, and capable of protecting bioactives during storage and simulated digestion. This study highlights three main contributions: (i) green extraction of chemically diverse compounds using supramolecular solvents, avoiding toxic reagents; (ii) efficient encapsulation of phytocomplexes into nutriosomes, yielding nanoscale, stable vesicles with high antioxidant retention abilities; and (iii) improved in vitro retention and release behavior, particularly under simulated gastrointestinal conditions, compared to conventional liposomes. Importantly, while these findings demonstrate a robust methodological platform for functional ingredient development, claims of nutritional or health-promoting effects remain beyond the scope of this study. Such claims require validation through biological activity assays, absorption studies, and in vivo trials. Therefore, this work should be considered as a proof-of-concept foundation for future translational research aimed at developing functional foods and nutraceutical applications.

## Figures and Tables

**Table 1:** Mean diameter, polydispersity index, zeta potential, and retention of antioxidant activity of liposomes and nutriosomes loading the phytocomplexes from Sango radish and kale.

	Nutriosome		Liposome	
	Sango radish	Kale	Sango radish	Kale
Mean diameter (nm)	110 ± 4 ^a^	104 ± 3 ^ab^	102 ± 2 ^b^	99 ± 2 ^b^
Polydispersity index (au)	0.10 ± 0.01 ^b^	0.10 ± 0.01 ^b^	0.11 ± 0.02 ^b^	0.19 ± 0.03 ^a^
Zeta potential (mV)	−41 ± 2 ^ab^	−42 ± 3 ^a^	−36 ± 2 ^b^	−39 ± 3 ^ab^
Retention of antioxidant activity (%)	96 ± 1 ^ab^	99 ± 1 ^a^	91 ± 1 ^c^	94 ± 1 ^b^

Note: Mean values ± standard deviations are reported. Same superscripted letters indicate values that are not statistically different (*p* < 0.05).

**Table 2:** Physicochemical and color characteristics of freeze-dried liposomes and nutriosomes. Mean values ± standard deviations are reported. Same letters indicate values that are not statistically different (*p* < 0.05).

Type of Nanovesicles	Nutriosome		Liposome	
Parameter	Sango radish	Kale	Sango radish	Kale
Moisture content (g/100 g)	8.3 ± 0.0 ^a^	7.5 ± 0.0 ^c^	7.9 ± 0.0 ^b^	7.1 ± 0.0 ^d^
Solubility (g/100 g)	50 ± 0 ^d^	74 ± 0 ^b^	62 ± 0 ^c^	77 ± 0 ^a^
Hygroscopicity (g/100 g)	12 ± 0.00 ^a^	12 ± 0 ^a^	12 ± 0 ^c^	12 ± 0 ^b^
Bulk density (g/mL)	0.17 ± 0.00 ^ab^	0.17 ± 0.01 ^ab^	0.17 ± 0.00 ^ab^	0.16 ± 0.00 ^b^
Tapped density (g/mL)	0.22 ± 0.00 ^b^	0.21 ± 0.00 ^c^	0.31 ± 0.01 ^a^	0.29 ± 0.01 ^a^
Carr Index (%)	21 ± 0.1 ^b^	19 ± 0.1 ^c^	47 ± 0.3 ^a^	47 ± 0.5 ^a^
Hausner ratio	1.3 ± 0.0 ^b^	1.2 ± 0.0 ^c^	1.9 ± 0.0 ^a^	1.9 ± 0.0 ^a^
Flowability	Fair	Good	Very bad	Very bad
Cohesiveness	Intermediate	Intermediate	High	High
Color				
L* (lightness)	80 ± 0.0 ^d^	93 ± 0.0 ^a^	88 ± 0.0 ^c^	93 ± 0.0 ^b^
a* (redness/greenness)	−0.7 ± 0.0 ^a^	−4.6 ± 0.0 ^c^	−4.4 ± 0.0 ^b^	−4.8 ± 0.0 ^d^
b* (yellowness)	23 ± 0.1 ^b^	28 ± 0.1 ^a^	19 ± 0.1 ^c^	22 ± 0.1 ^b^
C* (saturation)	23 ± 0.0 ^b^	28 ± 0.0 ^a^	20 ± 0.0 ^c^	22 ± 0.0 ^b^

**Table 3:** Microbiological quality of freeze-dried vesicle formulations during storage.

Test Sample	Total Aerobic Bacteria (log CFU/g or mL)	Enterobacteriaceae (log CFU/g or mL)	Yeasts and Fungi (log CFU/g or mL)	*Salmonella* spp. (Presence/Absence)	Sulfite-Reducing Clostridia (log CFU/g or mL)
Kale upper-phase	<1	<1	<1	/ *	/
Kale lower-phase	<1	<1	<1	/	/
Sango radish upper-phase	<1	<1	<1	/	/
Sango radish lower-phase	<1	<1	<1	/	/
Kale liposomes	<1	<1	<1	absence	<1
Sango radish liposomes	<1	<1	<1	absence	<1
Kale nutrisomes	<1	<1	<1	absence	<1
Sango radish nutrisomes	<1	<1	<1	absence	<1

* Analysis was not included in the panel for the sample.

## Data Availability

The original findings and contributions of this study are detailed within this article. For additional information, please contact the corresponding authors.

## Supplementary Materials

The following supporting information can be downloaded at: https://www.mdpi.com/article/10.3390/molecules30183774/s1, Table S1: Calibration curve information for phenolic acids and carotenoids.
