# Supplementary material for: Sustainable Supramolecular Extraction of Phytocomplexes from Microgreens and Their Eco-Loading in Nutriosomes: Physicochemical Characterization, Stability, and In Vitro Release Behavior
Source: Molecules. 2025 Sep 17;30(18):3774. doi: 10.3390/molecules30183774 (PMC12472398; doi:10.3390/molecules30183774)
Supplement: Supplementary file 1 [file molecules-30-03774-s001.zip › molecules-3837800-supplementary.pdf]

Supplementary Table S1. Calibration curve information for phenolic acids and carotenoids

Compound	Calibration equation (y = ax + b)	r^2
Syringic acid	y = 5603.5x + 3.5833	0.997
Gallic acid	y = 5190x − 2.5022	0.9823
Ferulic acid	y = 17.508x + 3.4013	0.9879
Rosmarinic acid	y = 416.84x + 0.1837	0.9535
Ellagic acid	y = 1682.6x + 8.2674	0.9875
Sinapic acid	y = 5402.1x + 0.2912	0.9996
Gentisic acid	y = 3503.3x − 3.473	0.9745
Lutein	y = 9301.6x − 0.1033	0.9982
Zeaxanthin	y = 9301.6x − 0.1033	0.9982
β-Carotene	y = 25101x − 7.8468	0.9711
